# Biomechanical and histological comparison between the cryopreserved and the lyophilized Gracilis tendon allograft for MPFL reconstruction, a cadaveric experimental study

**DOI:** 10.1186/s40634-016-0056-2

**Published:** 2016-09-06

**Authors:** Roberto Negrín, Jaime Duboy, Fernando Olavarría, Mauricio Wainer, Horacio Jimenez, Facundo Las Heras, Nicolas Reyes, Hugo Godoy

**Affiliations:** 1 Department of Orthopaedics and Traumatology, Clinica Las Condes, Lo Fontecilla 196, Santiago, 7591018 Chile; 2 Department of Pathology, Clinica Las Condes, Lo Fontecilla 196, Santiago, 7591018 Chile

**Keywords:** Cryopreserved Gracilis tendon allograft, Lyophilized Gracilis tendon allograft, Medial patellofemoral ligament, Maximum tensile force, Patellofemoral instability

## Abstract

**Background:**

Medial patellofemoral ligament (MPFL) is the main restrictor of lateral shifting of the patella, contributing by 60 % in the first 20° flexion of the knee. MPFL reconstruction has been performed in order to restore the stability of the patella with good results.Lyophilized Gracilis tendon allograft (LGA) compared to Cryopreserved Gracilis tendon allograft (CGA) has a lower cost, does not require to maintain cooling chain or preparation. The purpose of this study is to compare the histological and biomechanical characteristics of an experimental model of reconstruction of the MPFL in porcine patellas with LGA versus CGA.

**Methods:**

Randomized controlled experimental study in porcine model conducted on 36 porcine patellas in which 18 were intervened with LGA and 18 were intervened with CGA. The confluent tunnel technique was used for MPFL reconstruction. Maximum tensile force, allograft elongation and stiffness of the construct were measured. The cellularity and collagen tissue distribution were evaluated in the allografts. The histological and biomechanical characteristics of the LGA were compared to those of the CGA.

**Results:**

The median of the maximum tensile force for the LGA group was 299.63 N and 280.86 N for the CGA group (*p* = 0.45). The median of the stiffness was 57.86 N/mm for the LGA and 54.23 N/mm for the CGA (*p* = 0.2). The median of the elongation for the LGA was 5.95 mm and 6.12 mm for the CGA (*p* = 0,29). The bone bridge failed in 88.88 % of the constructs with LGA and 94.44 % in those with CGA (*p* = 0.5).

**Conclusions:**

No differences were observed between the LGA group and the CGA group in maximum tensile force, elongation, stiffness, site of rupture and histological characteristics. The use of a lyophilized Gracilis tendon allograft for MPFL reconstruction confers the same histological and biomechanical characteristics as a cryopreserved Gracilis tendon allograft.

## Background

Traumatic dislocation of the patella may occur in healthy knees as well as within the context of patellofemoral instability, and recurrent dislocation is a frequent problem in young active patients. The medial patellofemoral ligament (MPFL) is the main restrictor of lateral shifting of the patella, contributing by 60 % in the first 20° flexion of the knee (Desio et al. 1998), and it has been observed that injury to MPFL occurs in 96 % of traumatic dislocation cases of the patella (Sallay et al. 1996). Conservative treatment has shown recurrence rates of 15–44 % (Nomura et al. 2002; Bitar et al. 2012). According to the data, the development of a procedure which restores the medial stability of the patellofemoral joint has become necessary.

In recent years, MPFL reconstruction has been performed in order to restore the stability of the patella with good results, in terms of stability recovery, in clinical trials and different surgical techniques have been used with this goal. The construction of confluent tunnels has been one of the most widely used techniques since it does not require costly medical supplies and it is a relatively easy technique to perform with satisfactory to excellent results in restoring stability (Ahmad et al. 2014; Li et al. 2014; Song et al. 2014; Reagan et al. 2015; Neri et al. 2015; Shah et al. 2012; Lippacher et al. 2014; Stupay et al. 2015). In the literature, this technique reports a 29 % complication rate compared to a 21 % rate with the suture technique. In spite of the latter, no technique is free of complications and the confluent tunnel technique presents a lower recurrence rate of instability than the suture technique (3.3 % vs. 4.8 %) (Shah et al. 2012).

For these purposes, the cryopreserved allografts are currently the most widely used and in our field, the cryopreserved Gracilis tendon allograft (CGA) is preferred. A new variety of allograft known as the lyophilized Gracilis tendon allograft (LGA) has recently been launched on the market, which unlike the cryopreserved Gracilis tendon allograft has a lower cost, does not require to maintain cooling chain nor does it require thorough preparation for its use. There are currently no research studies comparing the biomechanical differences between one graft and the other for medial patellofemoral ligament reconstruction (MPFL).

The objective of this study was to compare the structural properties of the reconstruction technique with confluent tunnels using two types of grafts, the lyophilized Gracilis tendon allograft versus the cryopreserved Gracilis tendon allograft in a porcine model. Our alternative hypothesis was that the lyophilized Gracilis tendon allograft presents similar histological and biomechanical characteristics for medial patellofemoral ligament reconstruction in comparison to the cryopreserved Gracilis tendon allograft.

## Methods

### Study groups and samples

A randomized controlled experimental study in a porcine model was conducted in which 36 fresh adults porcine patellas were used, from 100 kg, healthy male adults 2 years in average. All of these samples came from the same slaughterhouse and was labelled in its medial aspect with the corresponding laterality by the same veterinarian. The patellas height was in a range of 40–50 mm and a minimum width of 40 mm.

The experiment was conducted in the *Universidad de los Andes* material testing laboratory in December 2014. The porcine model was used given its similarity to young human bone proved in previous studies (Lee et al. 2012; Herbort et al. 2012).

Nine lyophilized Gracilis tendon allografts, maintained at room temperature, and 9 cryopreserved Gracilis tendon allografts, maintained in liquid nitrogen at temperatures between −40 and −90 °C, were used. The allografts were donated by MTF (*Musculoskeletal Transplant Foundation*) for research purposes and each one was divided in transverse section into two equal parts thus obtaining 18 lyophilized Gracilis tendon allografts and 18 cryopreserved Gracilis tendon allografts. Cut with blade doesn’t affect the results. The allografts where from 26 human males and 10 females. The allografts average original length was 23.99 cms and width of 5.03 mm (4–7 mm). The mean of the allografts assay length was 11.52 cms.

The inclusion criteria were: grafts with a minimum length of 21 cms and a minimum wide of 4 mm. The exclusion criteria were: anatomical part with macroscopic morphological alterations or in poor condition. All grafts were included in this study.

A selection of porcine patellas was made and divided into two groups. Each group had 9 left patellas and 9 right ones. The porcine patellas were subjected to blocked randomization in units of 4 for use and the 18 allografts in groups of three for use thus obtaining 2 groups of 18 patellas to be used in the lyophilized Gracilis tendon allograft group (experimental group) and 18 to be used in the cryopreserved Gracilis tendon allograft (control group).

The histological and biomechanical characteristics were compared between the experimental group (lyophilized Gracilis tendon allografts) and the control group (cryopreserved Gracilis tendon allografts). Each group was made up of 18 allografts and 18 porcine patellas.

The study was authorized by the *Clínica Las Condes* research ethics committee in compliance with the health regulation on the use of animals for scientific research purposes.

### Study procedure

**Fig 1:**
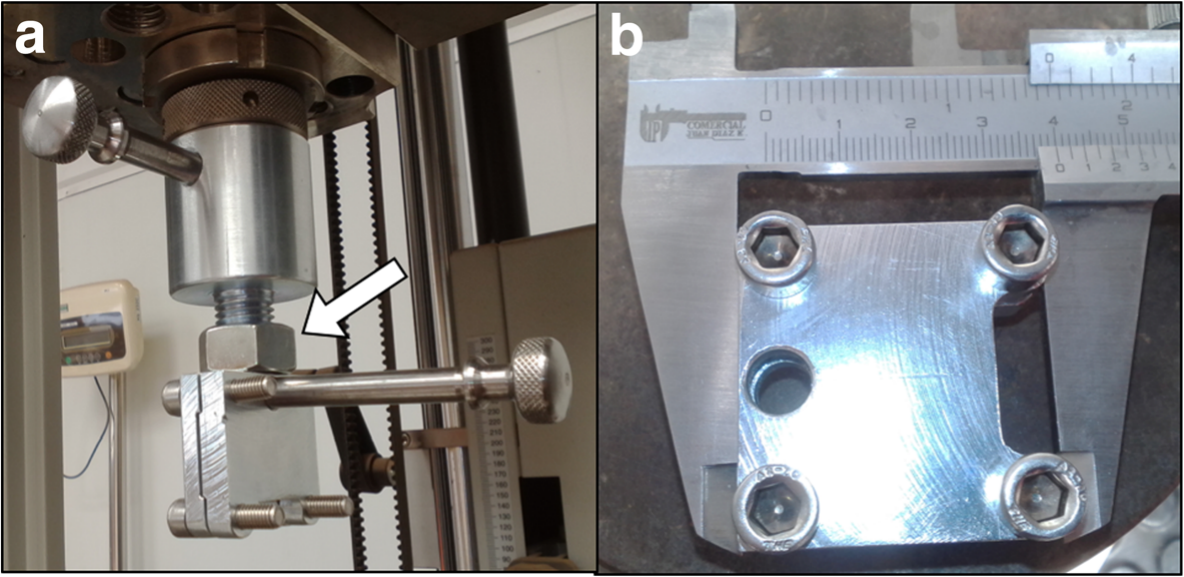
Testing Machine parts. **a** Adapter parts for the clamp attachment (*white arrow*) to the testing machine. **b** Allograft fixation clamp

The test was developed in a material testing laboratory with the same universal testing machine *Zwick Roell 300* ™ (Zwick Roell, Ulm, Germany). Adapter parts for the attachment of the clamp to the testing machine were developed (Fig. 1a). A hybrid fixation clamp model of stainless steel and a high resistance polymer (*ertacetal*) was used copying, with prior authorization by the authors, a clamp model tested to bear up to 2500 N tension without slippage of the tendon (Li et al. 2014) (Fig. 1b).

The procedure was developed by a single surgical team consisting of a surgeon with expertise in the development of the technique, two trauma surgeons and a general physician.

After being opened, each LGA was kept in sterile saline solution for a 15-min period and each CGA was kept in sterile saline solution for 15–20 min. A 1 cm long sample was obtained by transversal section from the less wide end of each allograft for the histological study, and afterwards it was sectioned into 2 parts each obtaining 18 LGA and 18 CGA. The free edges of the graft were prepared using reabsorbable sutures (*Vicryl* 2-0™, Ethicon, Cincinnati, USA) for fixation to the clamp during the trial.

**Fig 2:**
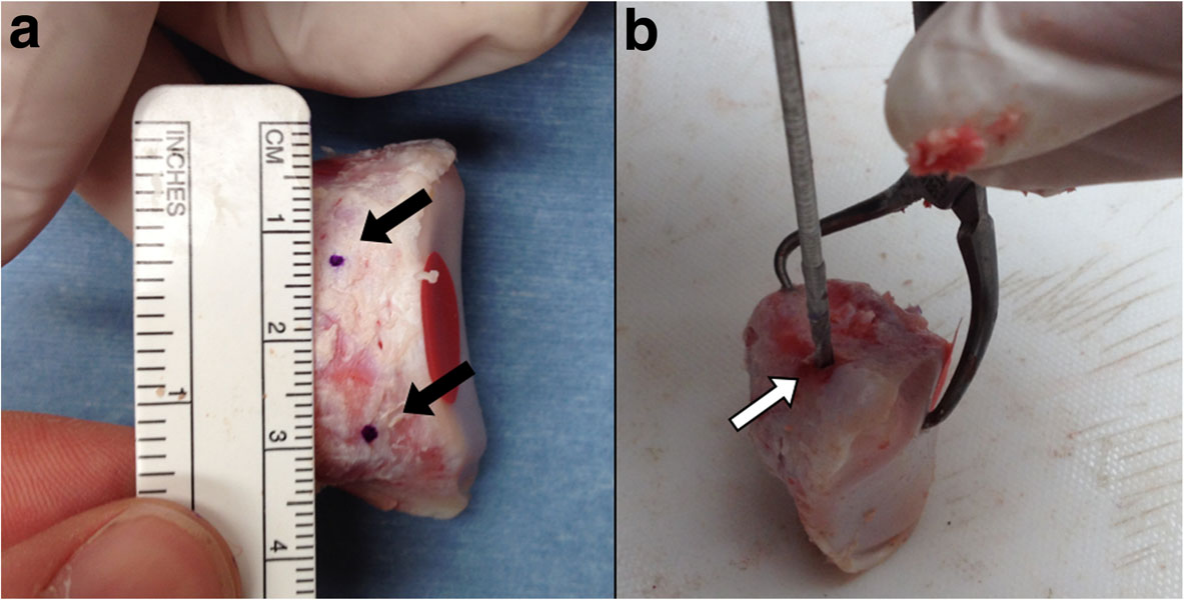
Patellar tunnels. **a** Position of confluent tunnels in medial aspect of the patella (*black arrows*), with 2 cms distance between each other. **b** Creation of axial tunnel for Steinmann pin (*white arrow*) which will be attached to the machine

The construction of the superior confluent tunnel was done in the medial aspect of the patella 1 cm below the superior edge and the inferior one, 2 cm from the superior one with an angle between both tunnels of approximately 90°. For this, first *K-needles* were used followed by 5 mm drills in order to construct the complete tunnel (Fig. 2a), model described by Ahmad et al. (2014).

The construction of a 5 mm diameter tunnel was performed, axial to the longitudinal axis of the slightly lateralized patella without communication with the confluent tunnels after which the Steinmann pin was installed in the universal testing machine (Fig. 2b).

Each of the patella/allograft units was constructed by passing the grafts through the tunnels using the traction of the sutures. Afterwards, equidistant length of the open ends of the tendon at the edge of the patella was achieved, with an average of 4.3 cm per end of which 2 cm were held in place by the clamp. The average length of free graft between the edge of the patella and the clamp was 2.3 cm, less than the regular mean distance of 5.5 cm of the LPFM in vivo (Shi et al. 2012).

The ends of the tendons were placed in the clamps through the traction of the sutures, applying n axial tension load in the following manner: first, 10 preload cycles in a load range between 5 and 20 N a 100 mm/min in order for the tendon to adjust, then 500 cycles of cyclic loads between 5 and 100 N, and finally, a maximum tension load of 200 mm/min until construct rupture. Elongation of the patella/allograft units was constantly measured.

A 1 cm section per graft was done for its histological analysis prior use in the biomechanical trial. The samples were analyzed using an *Olympus* (Olympus Corporation, Allentown, USA) optic microscope with 20× magnification. Digital photographs were taken using the software installed in the machine.

A *hematoxylin* and *eosin* stain was performed for cellularity analysis through a semiquanititative counting method of quadrants expressed in percentages. Additionally, a *Van Gieson* stain was carried out on different slices in order to differentiate the collagen fibers, which were described in a descriptive manner based on its disposition in: (1) dense collagen when the disposition was seen in barricade and (2) lamellar collagen when it observed to be less dense and with spaces between the fibers.

The evaluated results were: maximum tensile force, elongation, stiffness, elongation at the end of the load cycles and the site where the patella/allograft unit ruptured. Allograft cellularity and collagen disposition in the allografts were also evaluated. The manner in which the patella/allograft unit failed (bone bridge or tendon) was documented through digital photographs.

## Statistical analysis

The *Stata* 12.0 (StataCorp, College Station, USA) program was used to conduct the statistical analyses.

Nine unrelated samples of allografts were analized per group and 18 samples were included per group.

For the categorical variable description, absolute frequency and relative percentage were used, and for the continuous variables, minimum-maximum interval and median were used since they did not present normal distribution. The *Fischer* exact test was applied to compare the differences between the groups for the following variables: graft rupture site and collagen disposition. The non-parametric *Mann- Whitney* test was applied to compare the differences in maximum tensile force, final elongation, final elongation at the end of the cyclic loads and cellularity between the groups. A linear regression analysis was applied to the elongation and strength variables. Stiffness was calculated between 50 and 75 % of the maximum tensile force applied with the goal of proving the linear elongation in the interval corresponding to the elastic modulus of the tendon. Finally, the stiffness between the groups was compared through the *Mann – Whitney* non-parametric test. A statistical significance value of *p* <0.05 was established since a bilateral hypothesis was posited.

## Results

### Biomechanical results

**Fig. 3:**
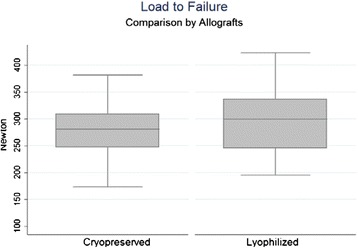
Maximum tensile force by allograft: LGA with median of maximum tensile force of 299.63 N (195.12–423.42 N) and CGA 280.86 N (172.77–381.27 N). No statistical differences (*p* = 0.45)

The median of maximum tensile force was 299.63 N (range 195.12–423.42 N) for LGA and 280.86 N (range 172.77–381.27 N) for CGA without statistical differences between the groups (*p* = 0.45) (Fig. 3). The median of maximum tensile force of all the allografts was 290.95 N. Two patella/allograft constructs of the LGA group and one of the CGA group, showed a maximum tensile force below 208 N.

**Fig. 4:**
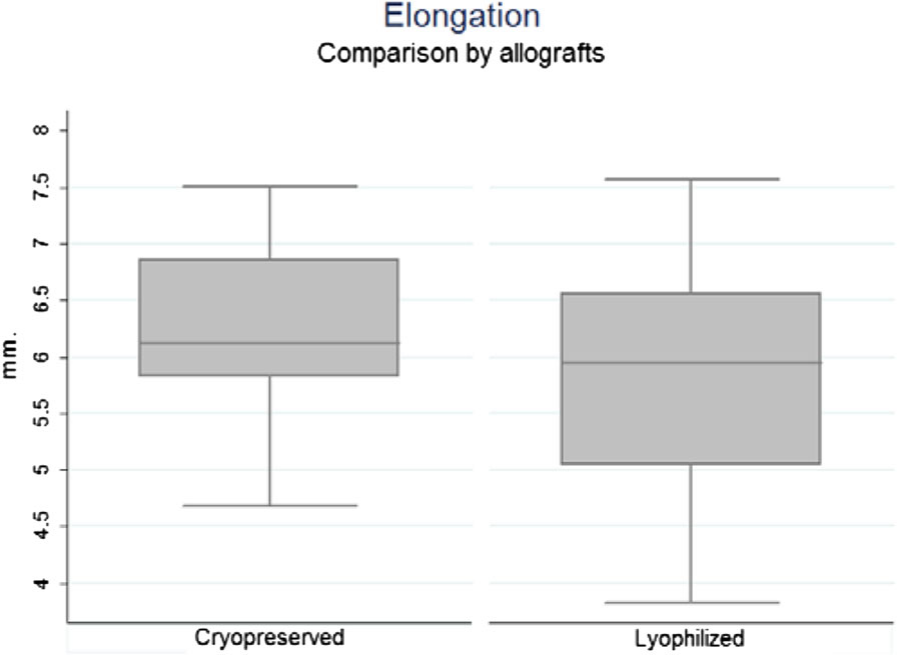
Elongation in maximum tensile force by allograft: LGA had a median elongation of 5.95 mm (3.83–7.57 mm) during maximum load of the allograft, and CGA had 6.12 mm (4.68–7.51 mm). No statistical differences (*p* = 0.29)

A median elongation of 5.95 mm was observed during maximum load of the allograft (range 3.83–7.57 mm) for LGA and 6.12 mm (range 4.68–7.51 mm) for CGA (*p* = 0.29) without statistical differences between the groups (*p* = 0.29) (Fig. 4). Median elongation observed in maximum load for all allografts was 6.03 mm.

**Fig. 5:**
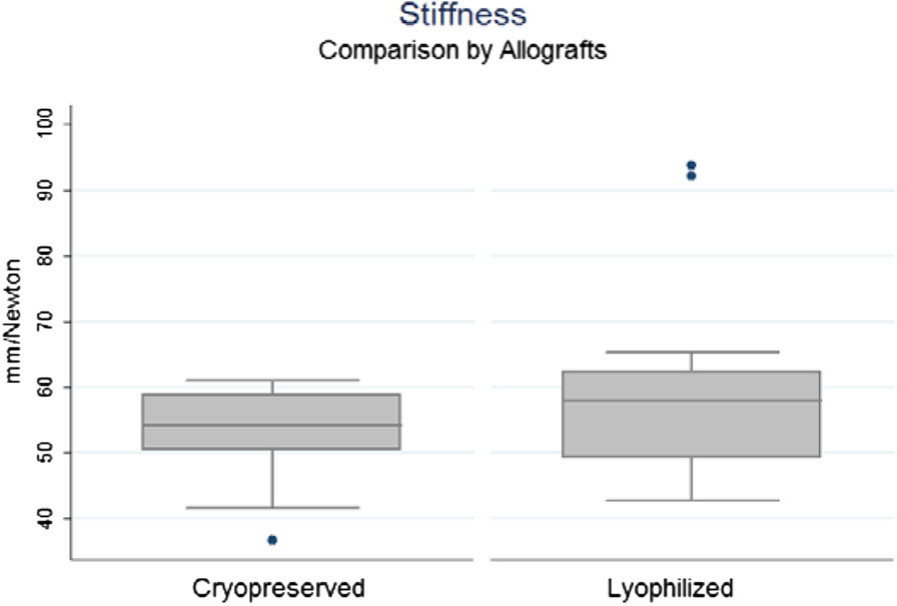
Stiffness by allograft: LGA had a median stiffness between 50 and 75 % of the maximum load applied calculated in 57.86 N/mm (42.68–93.69 N/mm), and CGA in 54.23 N/mm (36.66–61.02 N/mm) in the. No statistical differences (*p* = 0.2)

Median stiffness calculated between 50 and 75 % of the maximum load applied was 57.86 N/mm (range 42.68 93.69 N/mm) in the LGA and 54.23 N/mm (range 36.66–61.02 N/mm) in the CGA with no statistical differences being observed between the groups (*p* = 0.2) (Fig. 5). Linear regression calculated on a 50–75 % range of maximum load showed a high linearity (R^2^ > 0.98) in 36 out of 38 tests (94.4 %).

**Fig. 6:**
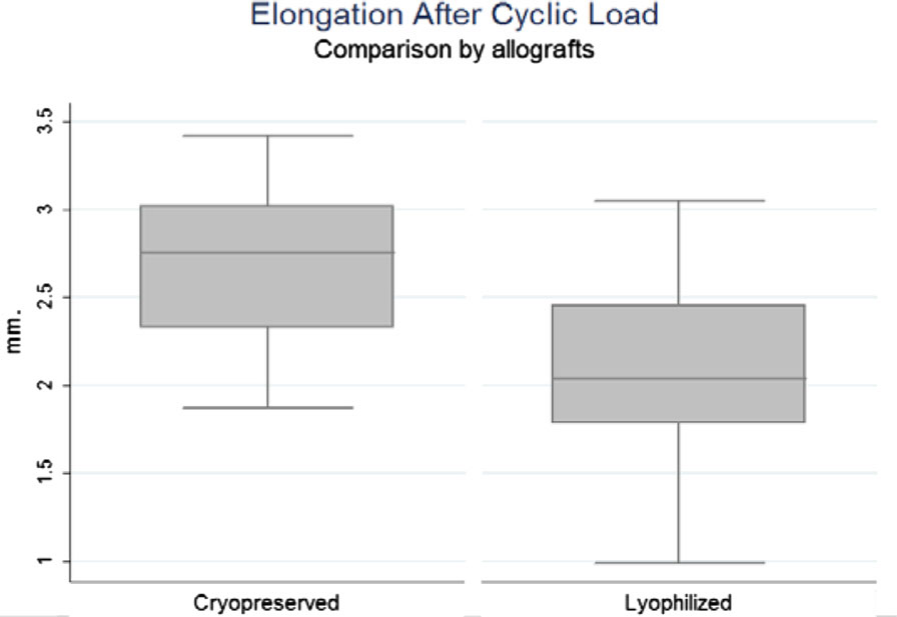
Elongation after cyclic load: A median allograft elongation for CGA of 2.66 mm (1.87–3.42 mm) after 500 load cycles, being higher than 2.09 mm (0.99–3.05 mm) for LGA. (*p* < 0.05)

All the patella/allograft units carried out the cyclic load test. A median allograft elongation of 2.66 mm (range 1.87–3.42 mm) after 500 load cycles in the CGA group, being higher in a statistically significant manner than the 2.09 mm (range 0.99–3.05 mm) observed in the LGA group (*p* < 0.05) (Fig. 6). A median elongation of 2.39 mm after cyclic loads was observed in all allografts.

**Fig. 7:**
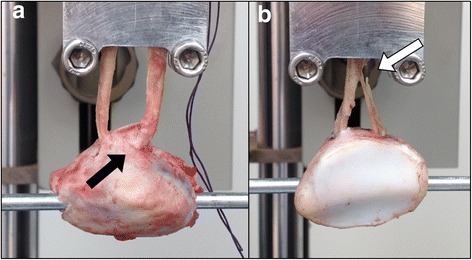
Failure sites. **a** Failure at bone bridge (*black arrow*). **b** Failure at allograft (*white arrow*)

Failure of the construct was not observed on any level after applying cyclic loads. After the application of maximum tensile strength, the patella/allograft unit failed at the bone bridge in 16 trials (88.88 %) (Fig. 7a) in the study group and in 17 trials (94.44 %) in the control group. The patella/allograft unitsfailed on an allograft level in 2 trials (11.12 %) in the study group and 1 trial (5.56 %) in the control group without statistical differences between the groups (*p* = 0.5). All the observed failures occurred close to the fixation clamp in both groups (Fig. 7b).

No differences were found between male and female specimens.

### Histological analysis

**Fig. 8:**
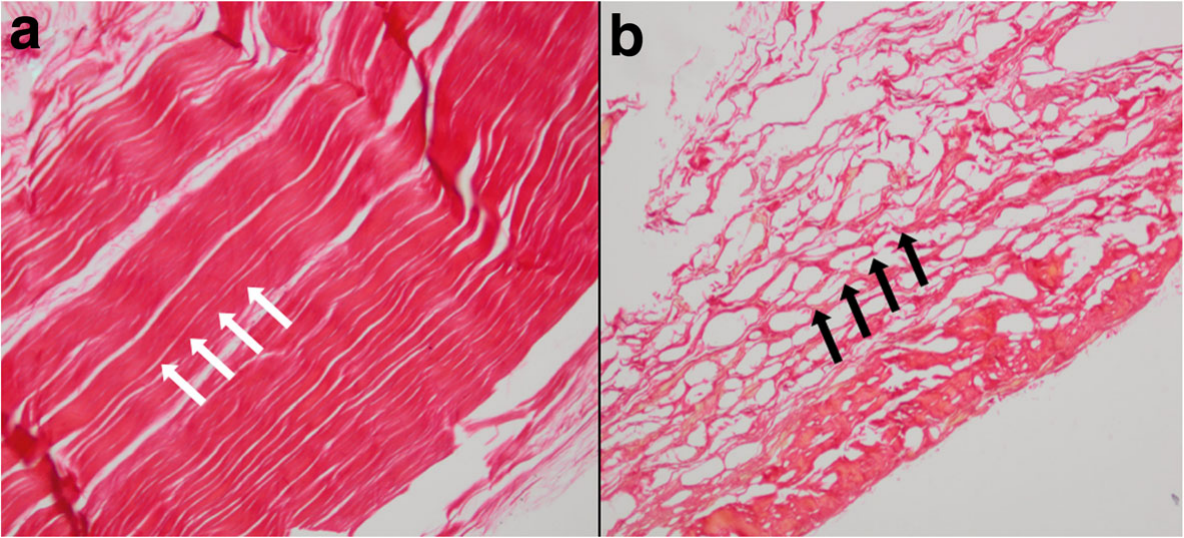
Allograft *Van Gieson stain*. Samples from one of the edges of each graft. **a** Barricade disposition of collagen (*black arrows*). **b** Lamellar disposition of collagen (*white arrows*)

The cellularity analysis showed values of 13.66 % for the lyophilized group and 9 % for the cryopreserved group without statistically significant differences between groups (*p* = 0.3). Mean cellularity observed was 13.3 % for both groups. The collagen analysis showed dense disposition in 55.6 % and 66.7 % (Fig. 8a), and lamellar in 44.4 and 33.3 % (Fig. 8b) of the samples for the lyophilized and cryopreserved groups respectively. Significant differences between the groups were not observed (*p* = 0.64).

## Discussion

Our study demonstrated the maximum tensile force born by the lyophilized allografts was 299.63 N (range 195.12–423.42 N), corresponding to a higher level than the native MPFL, calculated at 208 ± 90 N by Mountney et al. (2005). This value is similar to the 280.86 N (range 172.77–381.27 N) observed in the cryopreserved group and without statistical differences between the groups (*p* = 0.45). When observing the allografts that presented less resistance than the native MPFL (208 N) (Herbort et al. 2012), these did not show an aparent decreased density of the collagen disposition histologically evaluated in comparison to the rest of the study. Furthermore, the most of the sample in both groups failed in the bone bridge (anteromedial bone cortex). The porcine patellas measurements were similar to the humans patellas proportions as described by *Baldwin and House* in a total knee arthroplasty in vivo study (Baldwin and House 2005). The relative broad diameter of the patella tunnels (5 mm) posteriorly describe in the *Ahmad* et al. research (Ahmad et al. 2014) appears to be sufficient in relation to the 18 mm average of the medial patella thickness described by Baldwin & House (2005), but not in the stress conditions emulated in this experimental study. Smaller tunnels have to be probed in new experimental models to decrease the bridge bone failure rate even in detriment of the graft width.

Higuchi et al. (2010) observed through an evaluation in vivo with MRI, a total MPFL elongation between 0 and 120° of flexion, of 5 mm in healthy men, and 4 mm in healthy women. In our study, we obtained a 2.04 mm elongation at the end of the cycles (range 0.99–3.05 mm) in the LGA group and 2.75 mm (range 1.87–3.42 mm) in CGA group, less than what was observed in vivo, which could mean greater stiffness of the construct when applied in vivo in accordance with the *Lenschow* et al. findings (Lenschow et al. 2013).

Elongation in maximum tensile force, calculated before construct rupture showed values of 5.95 mm (range 3.83–7.57 mm range) for the LGA group and 6.12 mm (range 4.68–7.51 mm) for the CGA group, which exceeds the maximum elongation obtained by Higuchi et al. (2010). These results could simulate the critical conditions of elongation in MPFL reconstruction with the in vivo confluent tunnel technique thus positing it as a sufficient technique. In spite of this, the individuals evaluated by Higuchi et al. (2010) were patients who did not present knee pathology and henceforth does not reflect the altered biomechanics of patients with clinical patellofemoral instability. Micro CT for tendon-bone interfase was not possible, and it’s not needed for the objectives.

The rate of the histological area was not calculated. Every sample per allograft studied was 1 cm from the distal end. When conducting the comparison of collagen disposition between groups, statistically significant differences were not seen (*p* = 0.64), and the same proportion of lamellar and barricade collagen was observed.

When comparing cellularity, significant differences that would alter the biomechanical qualities of the grafts were not observed in the quantitative analyses.

Length of the grafts where not considered to be a determinant factor in outcomes in each group, because it was used a standardized method to cut and add to the construct, so the variations between each graft length were too small to add to the analysis.

We have no knowledge of biomechanical studies prior to this in which the biomechanical characteristics of these grafts were compared and consequently we consider the findings in favor of the use of the lyophilized Gracilis tendon allograft as an alternative for MPFL reconstruction to be important.

### Study limitations

The force vectors applied during the experimental set up of the study presented similar angles as to those observed in normal anatomy, however these angles are approximated and are not the same as those that result in live reconstruction in patients This may cause differences in the resistance of the graft and patellar bone bridge when comparing the results with studies in vivo.

The shape of a pig patella in its proximal third is narrower than the human one and therefore the proximal tunnel must be done 1 cm from the superior edge in order to avoid the construction of a shorter tunnel thus altering the original configuration described by Ahmad et al. (2014) which could decrease the comparability with studies in vivo.

The projection of the results, from study to clinical practice, must consider that the data obtained is determined by the structural characteristics of the construct in general and will only come close to those provided by a MPFL reconstruction in vivo.

## Conclusions

In our biomechanical study of MFPL reconstruction with Gracilis tendon allografts using the confluent tunnel technique, no difference in maximum tensile force, stiffness and elongation were observed between the groups. The control group (cryopreserved allografts) presented greater elongation after repetitive cyclic loads compared to the treatment group (lyophilized allografts). No differences were observed in cellularity and collagen disposition between groups.

From these results, we conclude that the maximum tensile force born by the allografts would be more than enough for MPFL reconstruction in vivo, and the use of lyophilized allografts would not pose a disadvantage, even showing a slightly superior resistance.
